# Inhibition of the 4-hydroxynonenal-regulated JNK/c-Jun pathway improves bleomycin-induced lung fibrosis

**DOI:** 10.1016/j.bj.2025.100916

**Published:** 2025-10-10

**Authors:** Chen-Chi Liu, Jiun-Han Lin, Tien-Wei Hsu, Chien-Ying Wang, Han-Shui Hsu

**Affiliations:** a Surgical Intensive Care Unit, Department of Critical Care Medicine, Taipei Veterans General Hospital, Taipei, Taiwan; b Faculty of Medicine, School of Medicine, National Yang Ming Chiao Tung University, Taipei, Taiwan; c Institute of Emergency and Critical Care Medicine, School of Medicine, National Yang Ming Chiao Tung University, Taipei, Taiwan; d Division of Thoracic Surgery, Department of Surgery, Taipei Veterans General Hospital, Taipei, Taiwan; e Department of Exercise and Health Sciences, University of Taipei, Taipei, Taiwan

**Keywords:** Pulmonary fibrosis, Lipid peroxidation, 4-HNE, JNK, TGF-β

## Abstract

**Background:**

Lipid peroxidation and 4-hydroxynonenal (4-HNE) contribute to oxidative stress-related tissue damage, but their roles in pulmonary fibrosis remain unclear. We examined their involvement in bleomycin-induced pulmonary fibrosis.

**Materials and methods:**

Lung fibrosis model mice were used to assess collagen deposition, lipid peroxidation markers, and oxidative stress. Ferroptosis inhibitors ferrostatin-1 (Fer-1) and deferoxamine (DFO) were administered to the mice. *In vitro*, murine lung epithelial (MLE-12) cells were treated with bleomycin, with or without lipid peroxidation inhibitors, and analyzed for oxidative stress and apoptosis. 4-HNE expression in idiopathic pulmonary fibrosis lung tissues was assessed using immunohistochemistry.

**Results:**

Bleomycin increased deposition of collagen and levels of 4-HNE and malondialdehyde levels while decreasing the glutathione/glutathione disulfide ratio. Fer-1 and DFO improved pulmonary function, reduced fibrosis, and restored the glutathione/glutathione disulfide ratio. *In vitro*, lipid peroxidation inhibition suppressed bleomycin-induced cell death and oxidative stress. Direct 4-HNE treatment induced apoptosis and lipid peroxidation, implicating 4-HNE in epithelial injury. 4-HNE upregulation was linked to increased transforming growth factor-β expression via c-Jun amino-terminal kinase/c-Jun signaling. Fer-1 and DFO mitigated these effects. Human idiopathic pulmonary fibrosis tissues exhibited elevated 4-HNE, correlating with fibrosis severity.

**Conclusions:**

Lipid peroxidation and 4-HNE play key roles in pulmonary fibrosis progression. Their regulation of transforming growth factor-β expression suggests targeting lipid peroxidation as a potential therapeutic strategy.

## Highlights

• 4-HNE and lipid peroxidation contribute to pulmonary fibrosis progression.
• Ferroptosis inhibitors reduce fibrosis by decreasing 4-HNE and oxidative stress.
• 4-HNE regulates TGF-β expression via JNK/c-Jun signaling in fibrosis.

## 1 Introduction

Idiopathic pulmonary fibrosis (IPF) manifests as a chronic progressive disease involving interstitial fibrosis of the lungs of unknown etiology, resulting in a substantial decline in pulmonary function over time [1]. Globally, it is estimated that around three million individuals are affected, and its incidence is dramatically correlated with aging [2]. Pathologically, IPF lungs are distinguished by aberrant accumulation of extracellular matrix constituents—most notably collagen—within alveolar and parenchymal regions. This process destroys the gas exchange surface and ultimately leads to respiratory insufficiency. The pathogenesis of IPF involves recurrent and subclinical epithelial injury, senescence of both alveolar epithelial cells and fibroblasts, and increased differentiation of fibroblasts into myofibroblasts [3]. However, the detailed mechanisms underlying these processes remain unclear.

Oxidative stress is an important mechanism leading to pulmonary fibrosis; previous studies indicated that elevated oxidative stress has been observed in IPF lungs and induces profibrotic mediator secretion while decreasing alveolar epithelial cell proliferation [4,5]. Among the downstream consequences of oxidative imbalance, lipid peroxidation stands out as a dominant and damaging event. The oxidative degradation of membrane lipids, notably polyunsaturated fatty acids, results in increased membrane permeability and reduced fluidity, ultimately impairing cell integrity. [6]. Furthermore, evidence indicates that lipid peroxidation in the lungs is associated with the occurrence and progression of pulmonary fibrosis [[7], [8], [9]].

Lipid peroxidation culminates in the generation of reactive aldehyde byproducts, notably 4-hydroxynonenal (4-HNE) and malondialdehyde (MDA), which serve as widely acknowledged proxies for oxidative stress, given their formation is tightly linked to reactive oxygen species–induced membrane damage [10]. In addition, 4-HNE exerts multifaceted biological activities by forming covalent adducts with proteins, thereby amplifying redox imbalance and modulating processes such as stress-responsive signaling, cell proliferation, and cell death. The pathological significance of 4-HNE extends across numerous disease contexts, including metabolic syndromes, neurodegeneration, and cancer [11]. However, the direct role of 4-HNE in the pathogenesis of pulmonary fibrosis, particularly through causal mechanisms in clinically relevant models, remains incompletely understood. Most existing studies remain correlative, lacking mechanistic clarity regarding its regulation of pro-fibrotic signaling pathways in the lung. Although consistent associations have been reported between 4-HNE and fibrotic diseases, direct causal evidence and mechanistic insights in clinically relevant models are still scarce. Our study addresses this gap by utilizing both bleomycin-induced fibrosis and human IPF tissue to define a mechanistic axis involving 4-HNE, JNK/c-Jun activation, and TGF-β induction.

Ferroptosis, a distinct modality of regulated cell demise contingent upon iron-dependent lipid peroxidation [12], is associated with pulmonary fibrosis [13,14], specifically exhibiting a link to high expression levels of 4-HNE and MDA. In contrast, glutathione (GSH), a critical suppressor of lipid peroxidation, is downregulated in the lungs of individuals diagnosed with IPF [15]. Ferrostatin-1 (Fer-1) and deferoxamine (DFO) are common ferroptosis inhibitors. Fer-1 functions as a radical-trapping antioxidant, impeding the accumulation of lipid hydroperoxides and thereby reducing the levels of reactive radicals [16,17]. DFO, a potent iron chelator, reduces the participation of Fe^2+^ in ferroptosis and has been used to treat diseases associated with iron overload [[18], [19], [20], [21]].

In this study, we explored the association between lipid peroxidation and pulmonary fibrosis and utilized Fer-1 and DFO to ameliorate bleomycin-induced pulmonary fibrosis. We further investigated the mechanistic link between 4-HNE and pro-fibrotic pathways, focusing on its regulation of transforming growth factor-β (TGF-β) expression via JNK/c-Jun signaling in both murine models and human IPF tissue. Our results provide direct evidence for a causative role of 4-HNE in pulmonary fibrogenesis and suggest that lipid peroxidation represents a viable target for therapeutic intervention in fibrotic lung diseases.

## 2 Materials and methods

### 2.1 Reagents

Bleomycin, Fer-1, DFO, *meta*-phosphoric acid, hydrocortisone, transferrin, insulin, β-estradiol, sodium selenite, l-glutamine, and HEPES were purchased from Sigma-Aldrich (St. Louis, MO, USA). Penicillin G and streptomycin were obtained from Corning, Inc. (Corning, NY, USA). 4-HNE and Rosiglitazone were obtained from MedChemExpress (Monmouth Junction, NJ, USA).

### 2.2 Patient lung tissue

Human lung specimens were acquired from individuals diagnosed with usual interstitial pneumonia who had undergone pulmonary resection procedures spanning the years 2000–2023. Normal lung tissues were derived from anatomically uninvolved regions excised during lobectomies performed for primary lung carcinoma. All experiments were approved by the Institutional Review Board of Taipei Veterans General Hospital (approval number: 2024-06-011AC).

### 2.3 Animal models

A bleomycin-induced lung fibrosis mouse model was used for IPF research. Male C57BL/6 mice aged six to seven weeks were purchased from BioLASCO Taiwan Co., Ltd. (Taipei, Taiwan). Pulmonary fibrosis was induced via a single intratracheal administration of 2 U/kg body weight of bleomycin (suspended in 50 μL of sterile phosphate-buffered saline). Control animals received the same volume of sterile PBS. To assess the effects of ferroptosis inhibition, Fer-1 (2.5 μmol/kg) [22] or DFO (100 mg/kg) [23] was dissolved in 200 μL saline and intraperitoneally administrated from days 1–7 to each animal treated with bleomycin. For ACSL4 inhibition, Rosiglitazone (0.5 mg/kg, dissolved in 200 μL saline) was administered intraperitoneally every other day for 14 days. Mice were euthanized on days 2, 4, 7, or 14 for further studies. All animal studies were conducted in accordance with the committee guidelines of the Institutional Animal Care and Use Committee of Taipei Veterans General Hospital (IACUC 2020-066).

### 2.4 Hematoxylin and eosin, Masson's trichrome, and Picro Sirius red staining

Mice lungs were fixed by 10 % neutral buffered formalin perfusion through intratracheal instillation and soaked in the same solution. After formalin fixation and paraffin embedding, lung blocks were cut into 4-μm-thick sections. These sections were stained with hematoxylin and eosin, Picro Sirius red (Sigma-Aldrich), and Masson's trichrome (Sigma-Aldrich). Areas containing collagen were stained as red by Picro Sirius red stain and blue by Masson's trichrome stain. Section images were quantified using an image-analysis system (Image-Pro Plus; Media Cybernetics, Silver Spring, MD, USA).

### 2.5 Immunohistochemistry

Adjacent serial sections were deparaffinized and rehydrated with xylene and different concentrations of ethanol. After antigen retrieval, the sections were blocked with hydrogen peroxide (3 %) and bovine serum albumin (1 %), and then incubated overnight with a polyclonal antibody specific to 4-HNE (1:800, ab48506, Abcam, Cambridge, UK), ACSL4 (1:500, ab155282, Abcam), and F4/80 (1:100, GTX26640, Genetex, Irvine, CA, USA) at 4 °C. Immunohistochemistry was performed with horseradish peroxidase-conjugated secondary antibodies (Dako, Carpinteria, CA, USA) for 30 min at room temperature. The samples were stained with 3,3′-diaminobenzidine chromogen solution and hematoxylin.

### 2.6 TUNEL assay

An *in situ* cell death detection POD Kit (Roche Molecular Biochemicals, Mannheim, Germany) was used to detect DNA fragmentation in cells according to the manufacturer's protocol. Briefly, the cells were fixed with 4 % paraformaldehyde and incubated with TUNEL solution in the dark for 60 min at 37 °C. Fluorescence images were obtained using an Olympus Provis AX70 microscope (Tokyo, Japan) equipped with a digital camera.

### 2.7 Immunofluorescence assay

Tissue sections were permeabilized with 0.1 % Triton X-100 (Sigma-Aldrich) for 30 min. After blocking with 1 % bovine serum albumin for 30 min, the slides were incubated overnight at 4 °C with primary antibodies against cytokeratin-18 (1:100, GTX105624, Genetex) or 4-HNE (1:25, GTX17571, Genetex) or with TUNEL assay reagent. The slides were washed and incubated with corresponding secondary antibodies, including goat anti-rabbit Alexa Fluor Plus 647 (1:100, ab150079, Abcam) and goat anti-mouse fluorescein isothiocyanate (1:100, ab97022, Abcam) for 1.5 h at room temperature. Finally, the slides were mounted with DAPI (Vector Laboratories, Burlingame, CA, USA). Images were acquired using a confocal microscope (FV3000, Olympus).

### 2.8 Western blot analysis

Tissue and cell lysates were collected in commercial lysis buffer (Corning, Inc.) containing protease inhibitor cocktail. Each sample was loaded into a 10 % sodium dodecyl sulfate-polyacrylamide gel, electrophoresed, and transferred onto polyvinylidene fluoride membrane (PerkinElmer Life and Analytical Sciences, Waltham, MA, USA). Membranes were blocked with 5 % milk (Bio-Rad, Hercules, CA, USA) in buffer composed of 20 mM Tris–HCl, 137 mM NaCl, and 1 % Tween 20 for 1 h at room temperature to prevent non-specific binding. The membranes were incubated overnight at 4 °C with primary antibodies against 4-HNE (ab48506, Abcam), p-c-Jun (Ser73) (ab30620, Abcam), c-Jun (9165, Cell signaling, Danvers, MA, USA), *p*-JNK (Thr183/Tyr185) (sc-293138, Santa Cruz Biotechnology, Dallas, TX, USA), JNK (sc-7345, Santa Cruz Biotechnology), ACSL4 (ab155282, Abcam), and Actin (20536, Proteintech, Rosemont, IL, USA). The membranes were incubated for 1 h at room temperature with horseradish peroxidase-labeled secondary antibody. The signals were recorded with a chemiluminescent system (PerkinElmer) and exposed to X-ray film to visualize the bands (Amersham Pharmacia Biotech, Inc., Piscataway, NJ, USA).

### 2.9 Measurement of lung function

Lung function tests were performed in unrestrained mice on day 0 before drug administration and on day 14 before sacrifice using barometric whole-body plethysmography (Buxco1; EMKA Technologies, Paris, France). The enhanced pause (Penh) values were calculated as an index of *in vivo* airway obstruction by Biopac AcqKnowledg software (BIOPAC, Goleta, CA, USA).

### 2.10 Hydroxyproline assay

Total lung hydroxyproline was measured using a hydroxyproline colorimetric assay kit (Abcam) according to the manufacturer's protocol. Briefly, lung tissue (10 mg) was homogenized in ddH_2_O (100 μL), mixed with 10 N NaOH (100 μL) at 120 °C for 1 h and then neutralized with HCl (100 μL). Next, the supernatant was transferred to a 96-well plate and evaporated in an oven set at 70 °C. Reaction reagents were added to each well, and absorbance was quantified at 560 nm using a SpectraMax M5 multi-mode reader (Molecular Devices, Sunnyvale, CA, USA)

### 2.11 MDA assay

MDA was measured with a Lipid Peroxidation Assay Kit (Abcam) according to the manufacturer's protocol. Briefly, tissues were homogenized in MDA Lysis Solution and, and the supernatant was collected. Thiobartbituric acid reagent was added to each supernatant and incubated at 95 °C for 60 min. After incubation, the mixtures were added to a 96-well microplate and absorbance was immediately measured at 532 nm using a SpectraMax M5 multi-mode reader.

### 2.12 GSH/GSSH assay

A GSH/glutathione disulfide (GSSG) detection kit (Enzo Life Sciences, Farmingdale, NY, USA) was utilized to determine reduced GSH and GSSG levels. Briefly, tissues and cells were homogenized with ice-cold 5 % *meta*-phosphoric acid and centrifuged. The supernatant was collected and mixed with GSH assay buffer. Absorbance in the 96-well plate was recorded at 405 or 414 nm using a plate reader at 1-min intervals over a 10-min period. The following equation was used for calculations: reduced GSH = total glutathione - oxidized GSSG.

### 2.13 Quantitative real-time PCR analysis

Total RNA was extracted from mouse lung samples and MLE-12 cells using an Illustra RNAspin mini kit (GE Healthcare, Little Chalfont, UK) according to the manufacturer's specifications. cDNA was synthesized by Arrow-Script Reverse transcriptase (ARROWTEC, Taipei, Taiwan). Relative primers were listed in [Supplementary Table S1]. Quantitative real-time PCR was performed using FastStart SYBR Green Master Mix (Roche Applied Science) and an ABI StepOnePlus Real-Time PCR System machine (Applied Biosystems, Foster City, CA, USA).

### 2.14 Cell cultures

Murine lung epithelial cells (MLE-12 cells) were obtained from ATCC (Manassas, VA, USA) and cultured in DMEM/F12 medium (Corning, Inc.) containing 4 % fetal bovine serum (Avantor, Inc., Radnor, PA, USA), 10 nM hydrocortisone, 0.01 mg/mL transferrin, 0.005 mg/mL insulin, 30 nM sodium selenite, 10 nM β-estradiol, 2 mM l-glutamine, 10 mM HEPES, 100 U/mL penicillin G, and 100 μg/mL streptomycin. A549 cells were obtained from ATCC (Manassas, VA, USA) and cultured in F–12K medium (Corning, Inc.) containing 10 % fetal bovine serum, 100 U/mL penicillin G, and 100 μg/mL streptomycin. The cells were incubated at 37 °C in a humidified atmosphere with 5 % CO_2_.

### 2.15 Cell viability assay

For viability assessment, 1 × 10^4^ cells per well were seeded into 96-well plates overnight. Next, the cells were treated with bleomycin or 4-HNE and incubated for 24 h. To the inhibitor groups, 100 μM DFO, 1 μM Fer-1, and 5 μM Rosiglitazone (Ro) were added. For the rescue of 4-HNE expression, cells were first treated with Ro and bleomycin for 8 h, followed by the addition of 4-HNE (40 μM) for 16 h. Subsequently, 10 μL of CCK-8 solution (Dojindo Laboratories, Kumamoto, Japan) was added and incubated at 37 °C for 1 h. After incubation, the plate was measured at 450 nm using a SpectraMax M5 multi-mode reader.

### 2.16 Lipid peroxidation detection with BODIPY 581/591 C11

Lipid peroxidation was assessed using C11-BODIPY 581/591 (D3861; Thermo Fisher Scientific, Waltham, MA, USA). The fluorescence emission peak was measured from 590 nm (red) to 510 nm (green) upon exposure to peroxyl radicals. MLE-12 cells were stained with 2 μM C11-BODIPY 581/591 for 30 min at 37 °C in the dark. They were then resuspended in 0.5 mL phosphate-buffered saline and evaluated using flow cytometry (FACS-Calibur®, Immunofluorometry Systems, BD Biosciences, Franklin Lakes, NJ, USA). The mean fluorescent signals were analyzed using FlowJo software (TreeStar, Ashland, OR, USA).

### 2.17 Measurement of TGF-β levels by ELISA

Cell culture medium was centrifuged at 400×*g* for 5 min at 4 °C to remove debris. Serum samples were also collected. TGF-β levels were quantified using a commercial ELISA kit (Elabscience, Wuhan, China) according to the manufacturer's instructions.

### 2.18 Chromatin immunoprecipitation (ChIP)

The chromatin immunoprecipitation (ChIP) assay was performed using the ChIP kit (ab500; Abcam) by the manufacturer's instructions. Briefly, cells were resuspended in Formaldehyde, lysed, and sheared using sonicator (Bioruptor, UCD-200; Diagenode, Seraing, Belgium) to achieve an optimal DNA fragment (200–1000 bp). For the ChIP assay, the samples were incubated overnight at 4 °C with rotation using an appropriate concentration of IgG or c-Jun antibody (9165, Cell signaling). On the following day, The antibody-protein-crosslinked DNA and the input DNA samples were subjected to proteinase K digestion and purified. The resulting DNA fragments were subsequently analyzed via qPCR. Three potential binding sites were selected for primer design, and data are shown for the site with the greatest statistical significance (mouse: 2184; human: 2055). Relative primers are listed in [Supplementary Table S2].

### 2.19 Statistical analysis

All data are reported as the means ± standard deviations. Student's two-tailed unpaired *t*-test or one-way ANOVA followed by Tukey's post-hoc test was employed. *p* < 0.05 were considered statistically significant.

## 3 Results

### 3.1 Lipid peroxidation and 4-HNE expression were increased in bleomycin-induced pulmonary fibrosis animal model

**Fig. 1:**
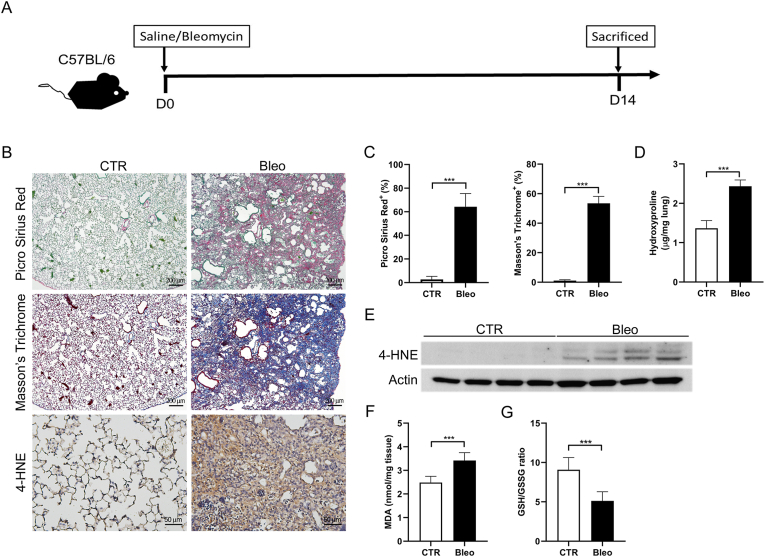
**4-HNE was upregulated in bleomycin-induced lung fibrosis animal model.** (A) Flowchart of experimental procedure. C57BL/6 mice were intratracheally administered 2 U/kg bleomycin (Bleo) or saline (CTR) on day 0. Mice were sacrificed on day 14, and lung tissues were stained with (B) Picro Sirius red or Masson's trichrome or subjected to and immunohistochemical staining to evaluate 4-HNE expression. Scale bars: 200 μm for Picro Sirius Red and Masson's Trichrome; 50 μm for IHC. (C) Quantification of Picro Sirius Red and Masson's trichrome staining. (D) Quantification of hydroxyproline. (E) Western blot analysis of 4-HNE, (F) malondialdehyde (MDA), and (G) GSH/GSSH ratio in mouse lung tissue. n = 6 per group. ∗∗∗*p* < 0.001.

To investigate whether lipid peroxidation plays an essential role in pulmonary fibrosis, we examined 4-HNE expression in a bleomycin-induced pulmonary fibrosis mouse model. We administered bleomycin or saline into the tracheobronchial tree of mice and examined changes in lung tissue after 14 days [Fig. 1A]. The bleomycin group showed an increased collagen content, as evidenced by Picro Sirius red and Masson's trichrome staining and high hydroxyproline levels [Fig. 1B–D]. In addition, immunohistochemistry and western blot analyses revealed increased 4-HNE expression in the bleomycin group (lower panel of [Fig. 1B and 1E]). Another lipid peroxidation marker, MDA, was elevated in the bleomycin-treated group [Fig. 1F]. In contrast, the GSH/GSSG ratio significantly decreased in the bleomycin group[Fig. 1G]. Moreover, longitudinal analysis demonstrated that 4-HNE levels increased in parallel with hydroxyproline content as fibrosis progressed over time [Supplementary Fig. S1A–S1C]. These data suggest that bleomycin induces lipid peroxidation and elevates 4-HNE expression in animal models of pulmonary fibrosis.

### 3.2 Inhibition of lipid peroxidation ameliorated severity of bleomycin-induced pulmonary fibrosis

**Fig. 2:**
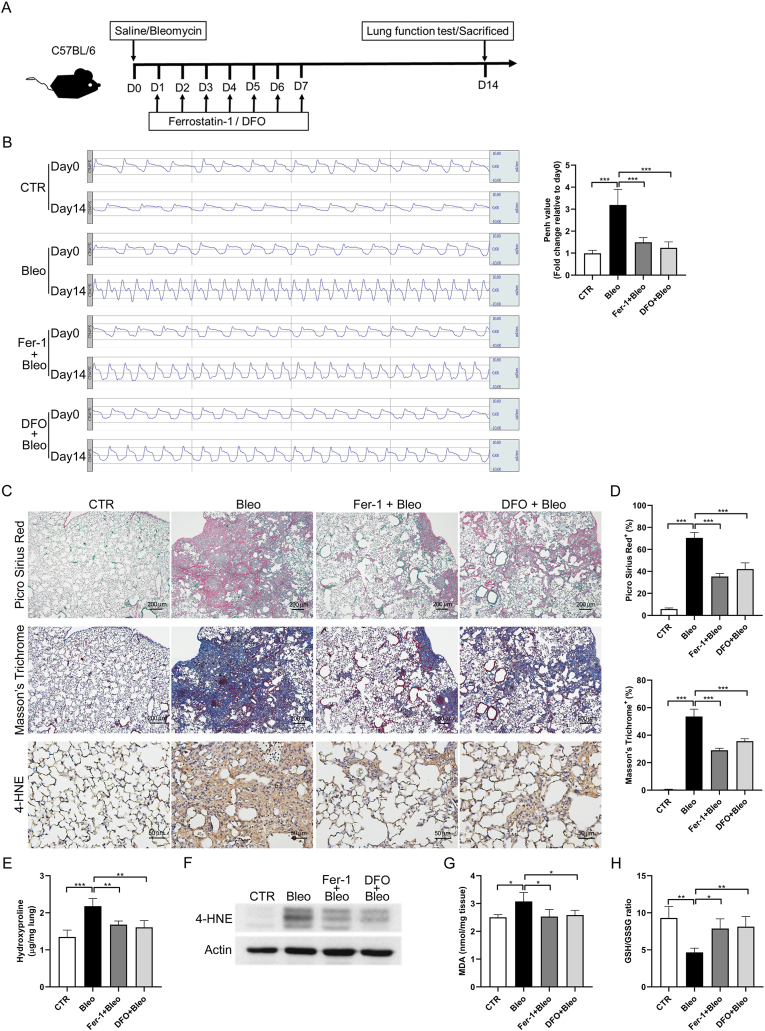
**Ferrostatin-1 (Fer-1) and deferoxamine (DFO) ameliorated bleomycin-induced pulmonary fibrosis by inhibiting lipid peroxidation and 4-HNE expression.** (A) Flowchart of experimental procedure. Mice with intratracheal instillation of bleomycin (Bleo, 2 U/kg) or saline (CTR) were treated with or without Fer-1 (2.5 μmol/kg) or DFO (100 mg/kg) from days 1–7 after bleomycin administration. (B) Barometric plethysmography was performed on day 0 before drug administration and on day 14. After sacrifice, mice lung tissues were subjected to (C) Picro Sirius red and Masson's trichrome staining and immunohistochemical staining of 4-HNE expression. Scale bars: 200 μm for Picro Sirius Red and Masson's Trichrome; 50 μm for IHC. (D) Quantification of Picro Sirius red and Masson's trichrome staining. (E) Hydroxyproline assay, (F) western blot analysis, (G) malondialdehyde (MDA) assay, and (H) glutathione (GSH)/glutathione disulfide (GSSH) ratio assay in mice lung tissues. n = 4–5 per group. ∗*p* < 0.05, ∗∗*p* < 0.01, ∗∗∗*p* < 0.001.

To investigate whether inhibition of lipid peroxidation can improve bleomycin-induced pulmonary fibrosis, we treated mice with Fer-1 or DFO from days 0–7 after bleomycin administration [Fig. 2A]. Pulmonary function was assessed using barometric plethysmography, revealing an improvement in mice treated with Fer-1 or DFO compared to those in the bleomycin group [Fig. 2B]. Fer-1 and DFO treatments also decreased collagen deposition and the degree of fibrosis induced by bleomycin [Fig. 2C–E]. Additionally, immunohistochemistry staining of the lung tissue and western blot analysis revealed that 4-HNE expression decreased after Fer-1 or DFO treatment (lower panel of [Fig. 2C and 2F]. Moreover, MDA levels were reduced and the GSH/GSSG ratio was increased after Fer-1 or DFO treatment compared to those in the bleomycin group [Fig. 2G and 2H]. These data suggest that inhibition of lipid peroxidation by Fer-1 or DFO reduced the severity of bleomycin-induced pulmonary fibrosis.

### 3.3 Fer-1 and DFO inhibited bleomycin-induced apoptosis and ferroptosis by reducing lipid peroxidation in murine lung epithelial cells

**Fig. 3:**
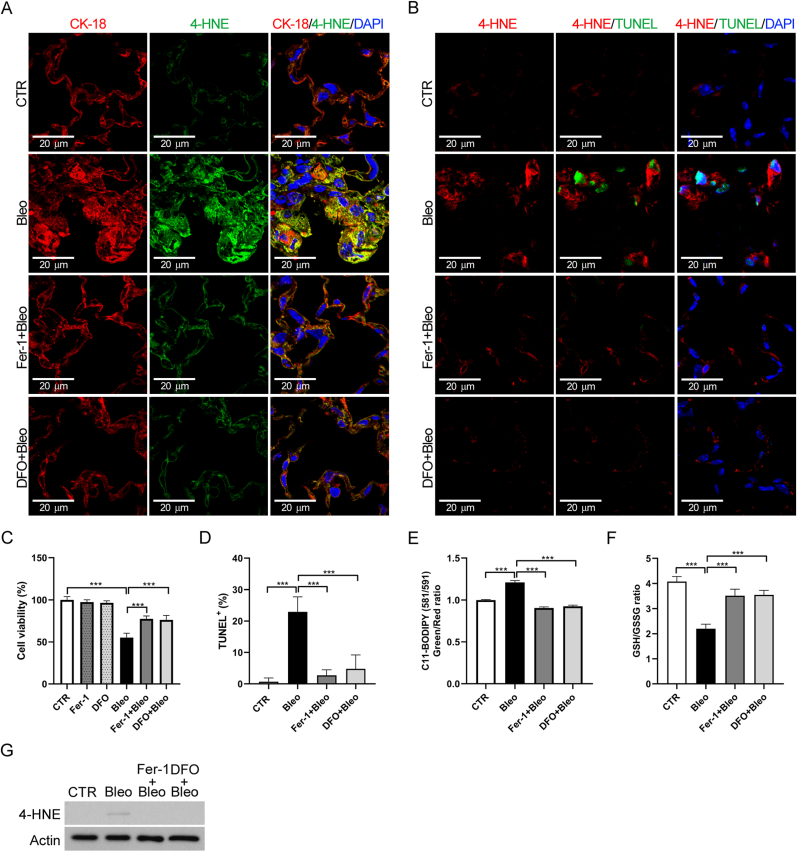
**Ferrostatin-1 (Fer-1) and deferoxamine (DFO) reduced bleomycin-induced apoptosis and ferroptosis in MLE-12 cells.** (A) Representative immunofluorescence images of mouse lung tissues showing co-staining with CK-18 (red) and 4-HNE (green). Cell nuclei were counterstained with DAPI (blue). (B) Representative immunofluorescence images of mouse lung tissues showing co-staining with 4-HNE (red) and apoptosis cells (green). Cell nuclei were counterstained with DAPI (blue). (C) Cell viability of MLE-12 cells treated with bleomycin (Bleo, 10 mU), Fer-1 (1 μM), and DFO (100 μM). (D) TUNEL assay to detect apoptosis. (E) Lipid peroxidation in MLE-12 cells was assessed by flow cytometry using C11-BODIPY after treatment with Bleo (10 mU), Fer-1 (1 μM), and DFO (100 μM). (F) GSH/GSSH ratio assay in MLE-12 cells treated with Bleo (10 mU), Fer-1 (1 μM), and DFO (100 μM). (G) Western blot analysis of 4-HNE expression in MLE-12 cells treated with Bleo (10 mU), Fer-1 (1 μM), and DFO (100 μM). ∗∗∗*p* < 0.001.

Injury to lung epithelial cells is a key factor in pulmonary fibrosis [24]. Immunofluorescence staining revealed that mouse lung epithelial cells (cytokeratin-18, a marker of alveolar epithelial cells) exhibited high 4-HNE expression after bleomycin-induced injury [Fig. 3A]. Furthermore, high 4-HNE expression was associated with apoptosis [Fig. 3B]. Notably, treatment with Fer-1 or DFO reduced 4-HNE expression and apoptosis in the lungs of the bleomycin model of IPF.

Because apoptosis of alveolar type II (AT2) pneumocytes is recognized as a key driver of pulmonary fibrosis, we selected MLE-12 cells, a murine AT2-derived epithelial cell line [25], to model epithelial injury. This choice allowed us to specifically examine how 4-HNE contributes to AT2 cell apoptosis and profibrotic signaling, thereby linking our findings to a cell population central to IPF pathogenesis. To evaluate the effect of lipid peroxidation on bleomycin-induced lung epithelial cell death, MLE-12 cells were treated with bleomycin in combination with Fer-1 or DFO for 24 h. Bleomycin induced cell death and apoptosis in MLE-12 cells; additionally, lipid peroxidation, increased 4-HNE expression, and a reduced GSH/GSSG ratio were observed. Treatment with Fer-1 and DFO inhibited cell death, lipid peroxidation, and 4-HNE expression and restored the GSH/GSSG ratio [Fig. 3C-G]. Similar results in terms of lipid peroxidation and TGF-β signaling were demonstrated in A549 cells [Supplementary Fig. S2], a human lung alveolar epithelial cell line. These data suggest that Fer-1 and DFO suppress bleomycin-induced apoptosis and ferroptosis by inhibiting lipid peroxidation in murine lung epithelial cells.

### 3.4 4-HNE induced apoptosis and ferroptosis in murine lung epithelial cells

**Fig. 4:**
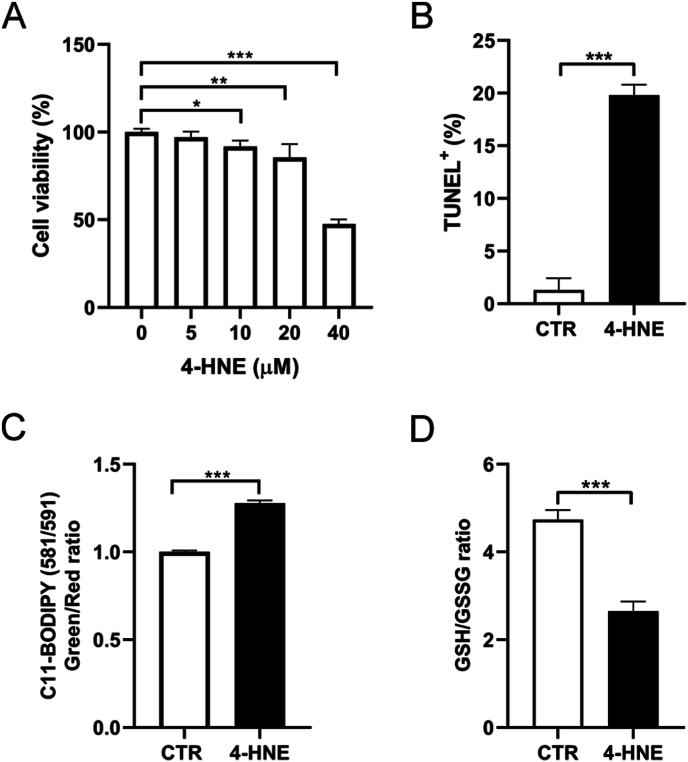
***In vitro* exposure to 4-HNE induced apoptosis and ferroptosis in MLE-12 cells.** (A) Cell viability of MLE-12 cells treated with indicated doses of 4-HNE. (B) TUNEL assay to detect apoptosis. (C) Lipid peroxidation in MLE-12 cells was assessed by flow cytometry using C11-BODIPY after treatment with 4-HNE (40 μM). (D) GSH/GSSH ratio assay in MLE-12 cells treated with 4-HNE (40 μM). ∗*p* < 0.05, ∗∗*p* < 0.01, ∗∗∗*p* < 0.001.

4-HNE induces ferroptosis and plays a role in lipid peroxidation [26]. To further explore the role of 4-HNE in pulmonary fibrosis, we treated MLE-12 cells with 4-HNE and examined cell viability, lipid peroxidation, and the GSH/GSSG ratio. Similar to the effects of bleomycin, 4-HNE induced cell death and apoptosis [Fig. 4A and 4B], promoted lipid peroxidation [Fig. 4C], and reduced the GSH/GSSG ratio [Fig. 4D]. Similar changes of cell viability and lipid peroxidation were demonstrated in A549 cells [Supplementary Fig. S3]. These findings suggest that 4-HNE accumulation leads to epithelial damage, thereby inducing pulmonary fibrosis.

### 3.5 4-HNE regulated TGF-β expression by activating the JNK/c-Jun pathway in bleomycin-induced pulmonary fibrosis

**Fig. 5:**
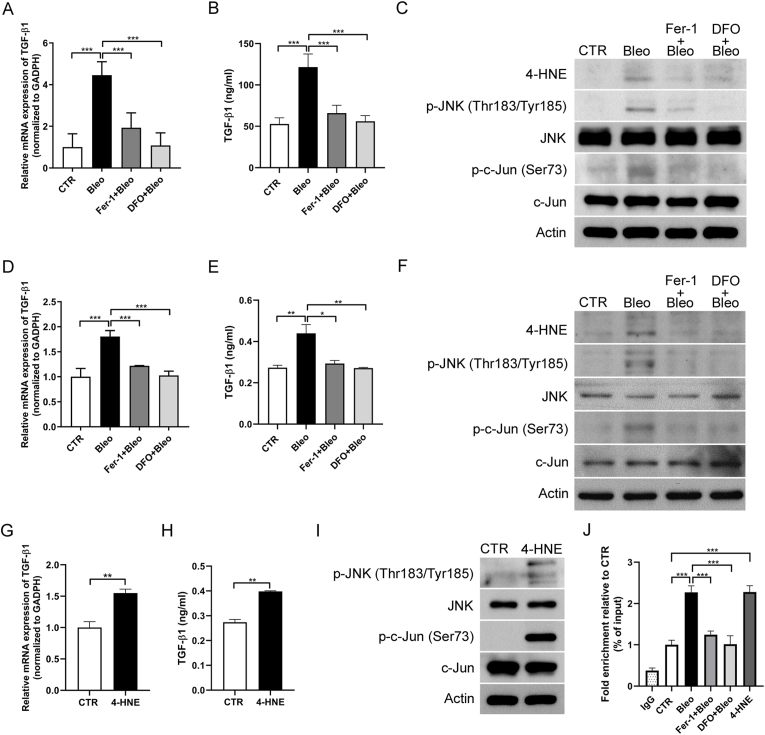
**4-HNE regulated TGF-β expression by activating JNK/c-Jun pathway both *in vivo* and *in vitro*.** (A) Relative mRNA expression of TGF-β, (B) Serum levels of TGF-β, and (C) western blot analysis of 4-HNE, phospho-JNK, and phospho-c-Jun in mice lung tissues after treatment with Bleo (2 U/kg), Fer-1 (2.5 μmol/kg), and DFO (100 mg/kg). n = 4 per group. (D) Relative mRNA expression of TGF-β, (E) TGF-β levels in the culture medium, and (F) western blot analysis of 4-HNE, phospho-JNK, and phospho-c-Jun in MLE-12 cells treated with Bleo (10 mU), Fer-1 (1 μM), and DFO (100 μM). (G) Relative mRNA expression of TGF-β, (H) TGF-β levels in the culture medium, and (I) western blot analysis of phospho-JNK and phospho-c-Jun in MLE-12 cells treated with 4-HNE (40 μM). (J) ChIP-qPCR analysis using a c-Jun antibody to assess protein–DNA binding at the TGF-β promoter in MLE-12 cells. Data were normalized to input and expressed relative to the CTR. ∗*p* < 0.05, ∗∗*p* < 0.01, ∗∗∗*p* < 0.001.

Pulmonary fibrosis is affected by various cytokines, among which TGF-β is the most potent stimulator of collagen production and pathogenesis of fibrosis [27,28]. We observed that bleomycin treatment increased 4-HNE expression in fibrotic lung tissue and MLE-12 cells, accompanied by elevated TGF-β mRNA levels in both lung tissue and cell lysates [Fig. 5A and 5D]; [Supplementary Fig. S4A], as well as increased TGF-β protein levels in serum and culture media [Fig. 5B and 5E]; [Supplementary Fig. S4B]. This effect was suppressed by Fer-1 or DFO. Direct 4-HNE exposure also increased TGF-β expression in MLE-12 and A549 cells [Fig. 5G and 5H]; [Supplementary Fig. S4D and S4E].

Additionally, activation of the JNK pathway enhances the expression of genes related to inflammation and fibrosis by phosphorylating c-Jun [29]. Studies also indicated that 4-HNE induces apoptosis by activating JNK and c-Jun [30,31]. To investigate the relationship between 4-HNE and JNK, we examined the expression of JNK signaling pathway proteins both *in vivo* and *in vitro*. Western blot analysis showed enhanced phosphorylation of JNK and c-Jun after bleomycin or 4-HNE treatment, which was inhibited by Fer-1 and DFO [Fig. 5C, 5F, 5I]; [Supplementary Fig. S4C and S4F]. Since 4-HNE can activate JNK/c-Jun and upregulate TGF-β, we investigate the relationship between c-JUN and TGF-β. A previous study has reported that c-Jun can directly bind to the promoter region of the TGF-β1 gene [32]. In line with this, we performed ChIP-qPCR analysis and confirmed that bleomycin and 4-HNE increased c-Jun binding to the TGF-β1 promoter [Fig. 5J]; [Supplementary Fig. S4G]. Together, these findings identify 4-HNE as a key activator of TGF-β expression through JNK/c-Jun signaling in pulmonary fibrosis.

### 3.6 ACSL4 inhibition attenuates bleomycin-induced pulmonary fibrosis by reducing 4-HNE–mediated JNK/c-Jun activation

**Fig. 6:**
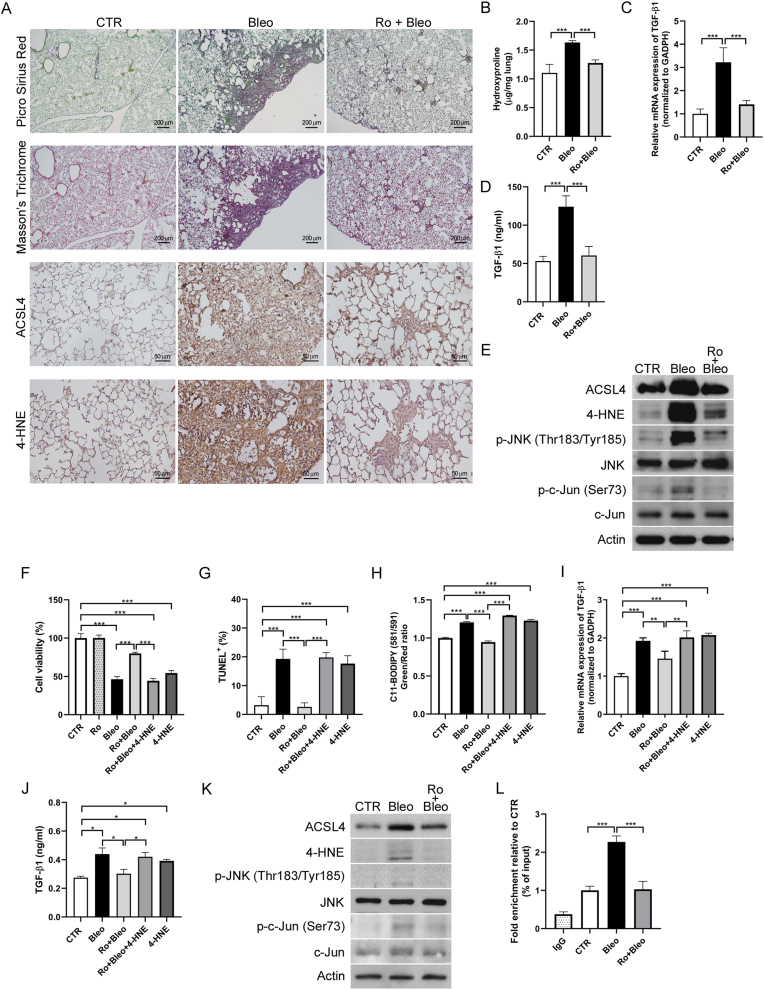
**ACSL4 inhibition attenuates bleomycin-induced pulmonary fibrosis by reducing 4-HNE and suppressing TGF-β via JNK/c-Jun activation.** *In vivo***:** Mice received intratracheal bleomycin (Bleo, 2 U/kg) or saline (CTR) and were treated with rosiglitazone (0.5 mg/kg, intraperitoneally) every other day for 14 days. (A) Picro Sirius red, Masson's trichrome, and immunohistochemical staining. Scale bars: 200 μm for Picro Sirius Red and Masson's Trichrome; 50 μm for IHC. (B) Hydroxyproline content. (C) Lung TGF-β mRNA expression. (D) Serum TGF-β levels. (E) Western blot analysis of lung tissue. *In vitro*: MLE-12 cells were treated with Bleo (10 mU), Ro (5 μM), and/or 4-HNE (40 μM), followed by analysis of: (F) Cell viability, (G) TUNEL assay, (H) Lipid peroxidation, (I) TGF-β mRNA expression, (J) TGF-β levels in the culture medium, (K) western blot, and (L) ChIP-qPCR for c-Jun binding at the TGF-β promoter. Data were normalized to input and expressed relative to the CTR. ∗*p* < 0.05, ∗∗*p* < 0.01, ∗∗∗*p* < 0.001.

Since ACSL4 is a critical driver of lipid peroxidation and 4-HNE production, we applied the selective ACSL4 inhibitor Rosiglitazone (Ro) to examine its impact on fibrosis progression and 4-HNE–regulated TGF-β expression through the JNK/c-Jun pathway. Treatment with Ro markedly reduced bleomycin-induced fibrosis severity and collagen deposition in mice [Fig. 6A and 6B], and decreased TGF-β levels in lung tissue and serum [Fig. 6C and 6D]. ACSL4 inhibition also reduced 4-HNE accumulation and suppressed JNK/c-Jun phosphorylation [Fig. 6E].

*In vitro*, Ro improved cell viability, reduced apoptosis, lipid peroxidation, and TGF-β expression in MLE-12 cells exposed to bleomycin, while 4-HNE supplementation reversed these effects [Fig. 6F–J]. Western blot and ChIP-qPCR confirmed that ACSL4 inhibition decreased ACSL4 and 4-HNE levels, suppressed JNK/c-Jun phosphorylation, and reduced c-Jun binding to the TGF-β1 promoter [Fig. 6K and 6L]. Similar results were observed in A549 cells [Supplementary Fig. S5].

Collectively, these results demonstrate that targeting ACSL4-mediated lipid peroxidation alleviates bleomycin-induced pulmonary fibrosis by suppressing 4-HNE accumulation and downstream JNK/c-Jun-driven TGF-β activation.

### 3.7 4-HNE contributes to ECM remodeling and immune cell infiltration in bleomycin-induced pulmonary fibrosis

**Fig. 7:**
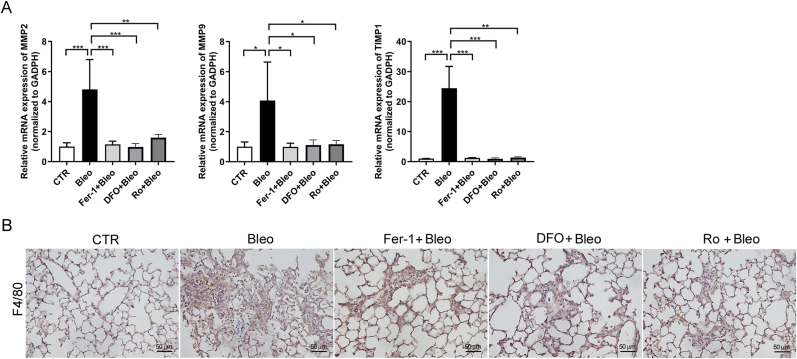
**Ferroptosis inhibition attenuates macrophage infiltration and extracellular matrix remodeling in bleomycin-induced lung fibrosis.** C57BL/6 mice were intratracheally administered saline (CTR) or bleomycin (Bleo) and treated with Fer-1, DFO, or rosiglitazone (Ro). (A) Relative mRNA expression of MMP2, MMP9, and TIMP1 in lung tissue. (B) Immunofluorescence staining of F4/80 to assess macrophage infiltration in lung tissue. Scale bars: 50 μm ∗*p* < 0.05, ∗∗*p* < 0.01, ∗∗∗*p* < 0.001.

To broaden the mechanistic scope of 4-HNE beyond TGF-β, we assessed extracellular matrix (ECM) remodeling by measuring MMP2, MMP9, and TIMP1, and evaluated immune cell infiltration using a macrophage marker, F4/80. We observed that bleomycin-induced upregulation of MMP2 and MMP9, as well as altered TIMP1 expression, was attenuated by Fer-1, DFO, or rosiglitazone treatment. Similarly, increased macrophage infiltration in fibrotic lungs was reduced following these treatments [Fig. 7]. These findings suggest that 4-HNE not only regulates TGF-β signaling but also contributes to ECM remodeling and immune cell recruitment in pulmonary fibrosis.

### 3.8 4-HNE was upregulated in IPF lung tissue

**Fig. 8:**
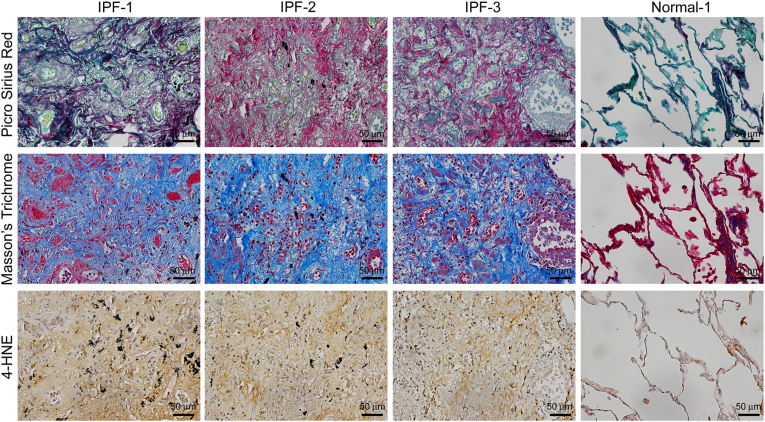
**4-HNE expression level was increased in the fibrotic area of IPF patients.** Picro Sirius red, Masson's trichrome, and IHC staining of 4-HNE expression in lung tissue of healthy controls and three patients with IPF. Scale bars: 200 μm for Picro Sirius Red and Masson's Trichrome; 50 μm for IHC.

We next examined whether 4-HNE expression was upregulated in human IPF lung tissue sections. Although our IPF lung tissues were derived from archival surgical specimens, this approach is consistent with prior studies such as Tsubouchi et al., which successfully identified lipid peroxidation markers in formalin-fixed paraffin-embedded samples [14]. Control tissues were carefully selected from non-tumor regions to minimize peritumoral inflammatory bias, ensuring relevance and reliability of biomarker analysis. Sirius Red and Masson's trichrome staining revealed elevated collagen levels in fibrotic sections. Moreover, the intensity of 4-HNE expression showed the same trend as the degree of fibrosis [Fig. 8] [Supplementary Fig. S6]. These results indicate that 4-HNE levels are increased in IPF, making it a possible therapeutic target.

## 4 Discussion

**Fig. 9:**
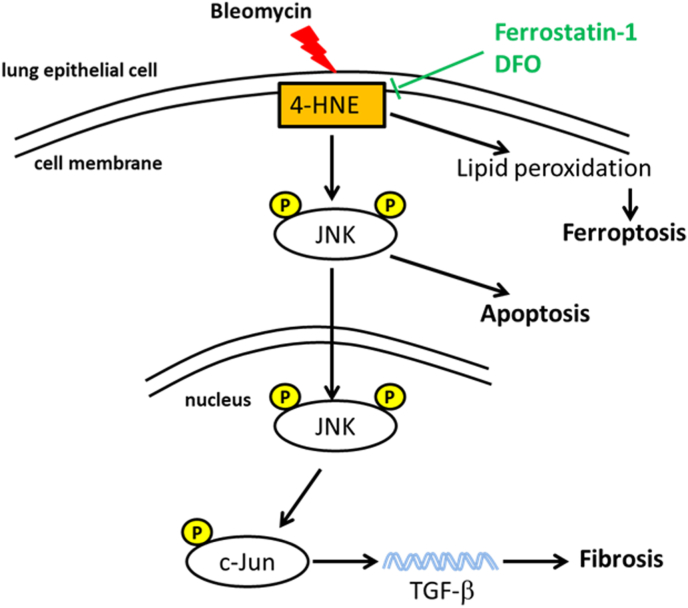
**Hypothetical model of 4-HNE in pulmonary fibrosis development.** 4-HNE induced epithelial damage and regulated TGF-β expression via JNK/c-Jun pathway activation, which can be inhibited by Fer-1 and DFO treatment.

During the global coronavirus disease (COVID-19) pandemic, a large number of severe pulmonary fibrosis cases were diagnosed, resulting in significant long-term effects. Pulmonary fibrosis is a severe and irreversible disease that causes various lung conditions. However, the effectiveness of the two available anti-fibrosis drugs (nintedanib and pirfenidone) is limited because they only slow the decline in lung function without halting disease progression [33]. Thus, effective anti-fibrotic strategies and therapies are urgently needed. Lipid peroxidation in the lungs is linked to the occurrence and progression of pulmonary fibrosis and serves as a hallmark of severity in patients with COVID-19. Specifically, 4-HNE has been associated with lethal outcomes in patients with aggressive COVID-19 [[7], [8], [9],^34^,^35^]. In this study, we demonstrated upregulation of lipid peroxidation and 4-HNE in IPF tissues and bleomycin-induced pulmonary fibrosis. We further investigated the underlying mechanism and found that 4-HNE triggered apoptosis in lung epithelial cells by activating the JNK/c-Jun signaling pathway. Suppressing the production of 4-HNE using the ferroptosis inhibitors Fer-1 and DFO improved lung function and alleviated pulmonary fibrosis. Additionally, these inhibitors reduced cell death and decreased the expression of TGF-β in lung epithelial cells. Taken together, these results indicate that 4-HNE plays an important role in pulmonary fibrosis [Fig. 9].

In addition to pulmonary diseases, chronic inflammation and oxidative stress have been implicated in the pathogenesis of cardiovascular disorders such as heart failure, myocardial infarction, and atherosclerosis. Systemic inflammatory mediators, including TNF-α and IL-6, are clinically used as diagnostic and prognostic markers in cardiovascular diseases and are known to activate fibrotic remodeling [36]. Similar to cardiac fibrosis, pulmonary fibrosis is characterized by inflammation-driven phenoconversion of resident cells into matrix-producing myofibroblasts. Studies have shown that cardiosome-derived microRNAs can promote this transition post-myocardial infarction [37], paralleling our findings that 4-HNE induces epithelial damage and TGF-β upregulation.

Moreover, post-translational modifications such as protein glycation also contribute to fibrotic outcomes in the cardiovascular system. Glycation of regulatory proteins like the ryanodine receptor has been associated with impaired cardiac response and worse prognosis [38]. These glycation events promote oxidative stress, inflammation, and fibrosis—similar to the downstream effects of 4-HNE. Therefore, it is plausible that both over-glycation and lipid peroxidation converge on shared profibrotic signaling cascades, such as the JNK-TGF-β axis, suggesting a common pathogenic mechanism in cardio-pulmonary fibrosis.

4-HNE, a downstream product of lipid peroxidation, exhibits high reactivity toward diverse biological macromolecules, including nucleic acids, lipids, and proteins. Additionally, previous findings indicate that 4-HNE can form adducts with DNA, leading to mutations and triggering apoptotic pathways [31,39]. These interactions, known as HNE–protein adduction, modify HNE activity. Owing to its propensity to generate such adducts and induce oxidative stress, 4-HNE has been recognized as a pivotal modulator of cellular processes, including oxidative stress signaling, cell proliferation, and cell death [11]. In addition, it serves as a central mediator of oxidative stress-related apoptosis and ferroptosis by affecting DNA, proteins, and lipids. During apoptosis, 4-HNE causes cell death by modifying proteins and DNA [40]. During ferroptosis, 4-HNE contributes to lipid peroxidation and antioxidant depletion, promoting a unique mode of cell death characterized by iron dependency and oxidative damage [41]. Understanding the dual role of 4-HNE in these processes may provide insights into therapeutic strategies for diseases associated with oxidative stress and cell death. Our data demonstrate that 4-HNE, similar to bleomycin, induces apoptosis and ferroptosis pathways within lung epithelial cells.

The lung epithelium is constantly subjected to stress, as evidenced by increased apoptosis in the lungs of patients with IPF [24,42,43]. Injured or stressed epithelial cells impair lung function, including gas exchange and fluid balance, and may trigger inflammatory responses by releasing signals that attract and activate immune cells. Moreover, these cells can produce TGF-β, a key mediator known to drive fibrotic process [44,45]. TGF-β promotes the fibrotic process through mechanisms such as epithelial-mesenchymal transition and extracellular matrix deposition. Additionally, TGF-β modulates the inflammatory response, which can exacerbate the fibrotic process [[46], [47], [48]]. Furthermore, 4-HNE may make an important contribution to upregulation of TGF-β1 expression [49]. We measured TGF-β RNA levels in fibrotic tissue and epithelial cells following treatment with bleomycin or 4-HNE. Our results demonstrate that bleomycin or 4-HNE-induced cell damage increased TGF-β RNA expression. Treatment with DFO or Fer-1 reduced cell injury and decreased TGF-β expression.

The JNK pathway is a critical mitogen-activated protein kinase pathway that conveys extracellular signals to cellular functions such as growth, migration, and programmed cell death [50]. JNK becomes activated through phosphorylation in response to environmental stress and inflammatory cytokines. Upon activation, JNK phosphorylates serine residues Ser63 and Ser73 in the N-terminal domain of c-Jun, modulating its activity [50,51]. Various stimuli implicated in both acute and chronic renal injury, such as alarmins, reactive oxygen species, cytokines, nephrotoxic agents, and fibrogenic signals, can activate the JNK pathway. JNK pathway activation can drive renal fibrosis by initiating inflammatory responses, apoptosis, and ferroptosis, as well as by enhancing TGF-β expression [29,52]. Notably, increased expression of active JNK has been observed in the epithelium of patients with IPF, which correlates with fibrosis progression [53]. Our data demonstrate that 4-HNE regulates TGF-β expression in lung epithelial cells in pulmonary fibrosis by upregulating the JNK/c-JUN pathway. Furthermore, inhibition of the JNK/c-Jun pathway by Fer-1 or DFO treatment reduced TGF-β expression, leading to improved lung function and reduced pulmonary fibrosis.

Our study contributes to this growing understanding by directly implicating 4-HNE as a mediator of epithelial injury and fibrosis through JNK/c-Jun-dependent TGF-β signaling. These findings emphasize the broader relevance of oxidative stress products across organ systems and underscore the need to target redox-activated fibrotic pathways for therapeutic development.

### 4.1 Experimental limitations

The bleomycin-induced pulmonary fibrosis mouse model is widely used and has provided critical mechanistic insights; however, it does not fully replicate the chronic, heterogeneous, and progressive course of IPF. Classic single-dose bleomycin injury produces an acute inflammatory phase followed by transient fibrotic remodeling, often with partial resolution, whereas human IPF is characterized by relentless progression, architectural distortion, and honeycombing. Outcomes also vary depending on strain, sex, age, and administration protocol. These differences necessitate cautious extrapolation of therapeutic efficacy from bleomycin to patients. Nonetheless, the reproducibility of epithelial injury and fibrogenic signaling makes the model valuable for probing disease mechanisms. We also acknowledge that relatively small sample sizes of mouse model may limit statistical power and generalizability. Following the Resource Equation approach [54], our group sizes are acceptable for mechanistic studies while minimizing animal use, but future work should include larger cohorts and formal power analyses. Importantly, in our study, we validated 4-HNE upregulation not only in bleomycin-exposed mice but also in human IPF tissues, thereby anchoring experimental findings to patient pathology and strengthening translational relevance.

Fer-1 and DFO were applied as experimental tools to suppress lipid peroxidation and clarify the pathogenic role of 4-HNE, rather than for pharmacokinetic or safety evaluation. While our study did not include a dose-response analysis for 4-HNE or the ferroptosis inhibitors Fer-1 and DFO, our primary objective was to elucidate the pathogenic role of 4-HNE in pulmonary fibrosis rather than to evaluate therapeutic safety or pharmacodynamics. In addition, the concentrations of Fer-1 and DFO used in our experiments were selected based on prior studies demonstrating effective inhibition of ferroptosis in various disease models, including neurotoxicity and ischemia-reperfusion injury [[55], [56], [57], [58]]. These doses ensured biological relevance and consistency with established protocols. Future investigations incorporating dose-response and toxicity profiling will be essential for translational applications. We also acknowledge that the clinical applicability of Fer-1 and DFO in IPF remains uncertain, as their safety, bioavailability, and efficacy in humans have not been tested. In this study, they were used as experimental tools to probe the pathogenic role of 4-HNE, and future translational work will be required to assess whether clinically viable agents targeting lipid peroxidation can provide therapeutic benefit.

We further recognize that murine models and in vitro systems cannot fully replicate the chronic and heterogeneous nature of human IPF. To address this limitation, we validated 4-HNE upregulation in human IPF tissues, thereby anchoring our mechanistic findings to patient pathology. However, the therapeutic implications remain preliminary and will require confirmation in longitudinal human studies and more advanced preclinical models. In addition, we focused on the JNK/c-Jun pathway because it consistently mediated 4-HNE–induced TGF-β upregulation in this study. It should also be noted that 4-HNE and lipid peroxidation may influence other pathways such as NF-κB and p38 MAPK, which were not investigated here but merit future exploration to fully delineate downstream mediators.

Due to the rarity of surgical intervention in IPF patients, access to human lung tissue is inherently limited. As a result, the sample size and associated clinical data in our study were constrained. Despite this limitation, our findings align with previous reports of elevated 4-HNE in fibrotic lungs, supporting its relevance. Future studies with larger cohorts and annotated clinical data will be necessary to enhance translational significance.

## 5 Conclusion

4-HNE was highly expressed in lung tissues from patients with IPF and in a bleomycin-induced animal model. Elevated 4-HNE levels contribute to epithelial damage and promote TGF-β expression. Mechanistically, 4-HNE regulates TGF-β expression through activation of the JNK/c-Jun pathway, which can be inhibited by Fer-1 and DFO treatments. These findings validate the therapeutic potential of 4-HNE in preventing and treating pulmonary fibrosis.

## Funding

This work was supported by the 10.13039/100020595National Science and Technology Council (NSCT 112-2326-B-039-001); 10.13039/501100011912Taipei Veterans General Hospital (V112C-014, V112C-106, V113C-149); and Lung Cancer Foundation, in memory of Dr. K. S. Lu, Taipei. We also extend our gratitude to the Memorial Foundation of Mr. Ching-Teh Hsu. The funding sources were not involved in study design, data collection, analysis, interpretation, report writing, or the decision to submit the article for publication.

## Declaration of competing interest

The authors declare no competing financial interests.

## Figures and Tables

**Figure:**
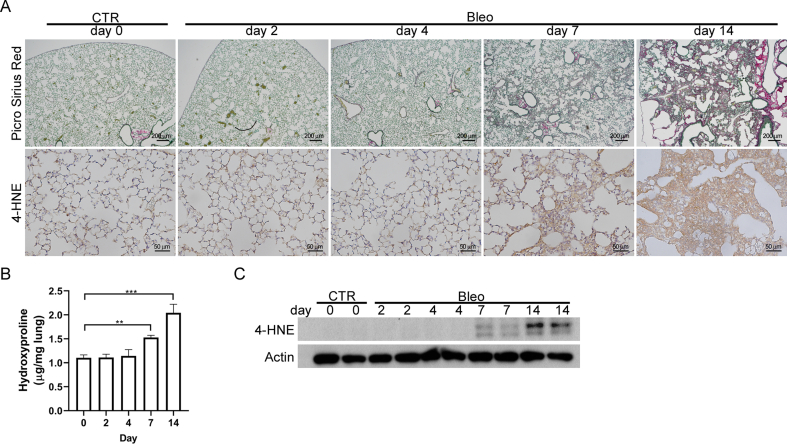
Supplementary Figure S1. **Collagen and 4-HNE are upregulated during the progression of bleomycin-induced lung fibrosis in mice.** C57BL/6 mice were intratracheally administered bleomycin (Bleo, 2 U/kg) or saline (CTR) on day 0 and sacrificed on days 2, 4, 7, and 14. Lung tissues were analyzed as follows: (A) Representative images of Picro Sirius Red and immunohistochemical staining of 4-HNE at days 0, 2, 4, 7, and 14. Scale bars: 200 μm (Picro Sirius Red), 50 μm (IHC). (B) Quantification of hydroxyproline content. (C) Western blot analysis of 4-HNE. n = 3 per group. ∗∗P < 0.01, ∗∗∗P < 0.001.

**Figure:**
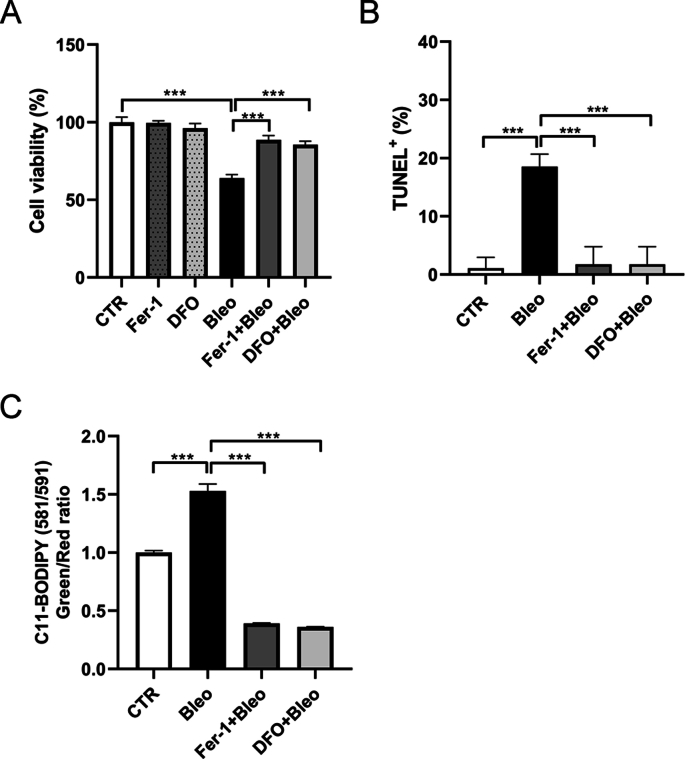
Supplementary Figure S2. **Ferrostatin-1 (Fer-1) and deferoxamine (DFO) reduced bleomycin-induced apoptosis and ferroptosis in A549 cells.** (A) Cell viability of A549 cells treated with bleomycin (Bleo, 10 mU), Fer-1 (1 μM), and DFO (100 μM). (B) TUNEL assay to detect apoptosis. (C) Lipid peroxidation in A549 cells was assessed by flow cytometry using C11-BODIPY after treatment with Bleo (10 mU), Fer-1 (1 μM), and DFO (100 μM). ∗∗∗P < 0.001.

**Figure:**
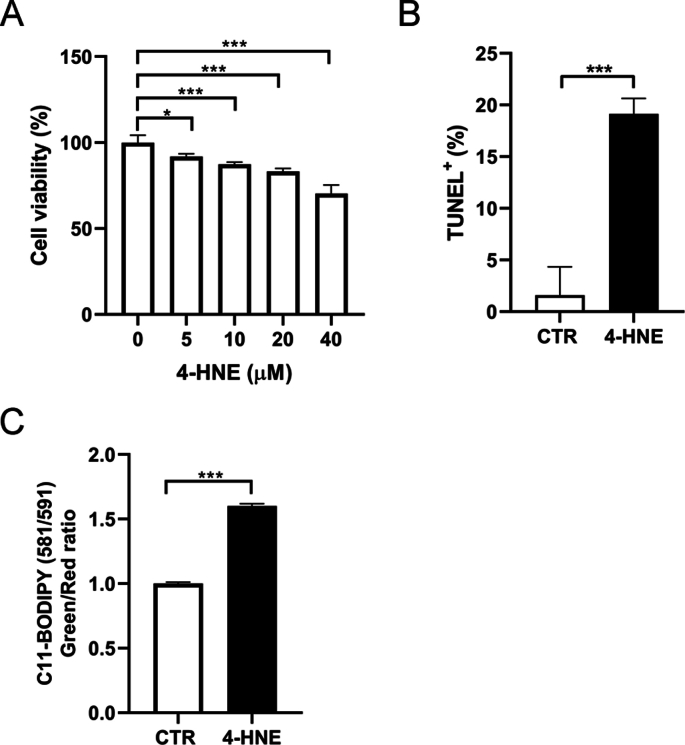
Supplementary Figure S3. ***In vitro*****exposure to 4-HNE induced apoptosis and ferroptosis in A549 cells.** (A) Cell viability of A549 cells treated with indicated doses of 4-HNE. (B) TUNEL assay to detect apoptosis. (C) Lipid peroxidation in A549 cells was assessed by flow cytometry using C11-BODIPY after treatment with 4-HNE (40 μM). ∗P < 0.05, ∗∗∗P < 0.001.

**Figure:**
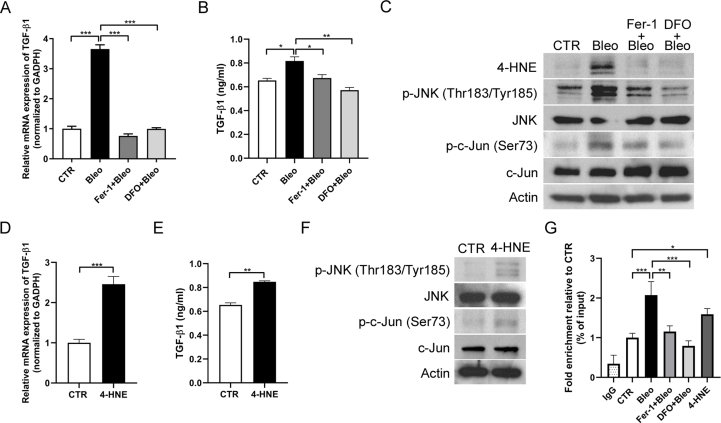
Supplementary Figure S4. **4-HNE regulated TGF-β expression by activating JNK/c-Jun pathway in A549 cells.** (A) Relative mRNA expression of TGF-β, (B) TGF-β levels in the culture medium, and (C) western blot analysis of 4-HNE, phospho-JNK, and phospho-c-Jun in A549 cells treated with Bleo (10 mU), Fer-1 (1 μM), and DFO (100 μM). (D) Relative mRNA expression of TGF-β, (E) TGF-β levels in the culture medium, and (F) western blot analysis of phospho-JNK and phospho-c-Jun in A549 cells treated with 4-HNE (40 μM). (G) ChIP-qPCR analysis using a c-Jun antibody to assess protein–DNA binding at the TGF-β promoter in A549 cells. Data were normalized to input and expressed relative to the CTR. ∗P < 0.05, ∗∗P < 0.01, ∗∗∗P < 0.001.

**Figure:**
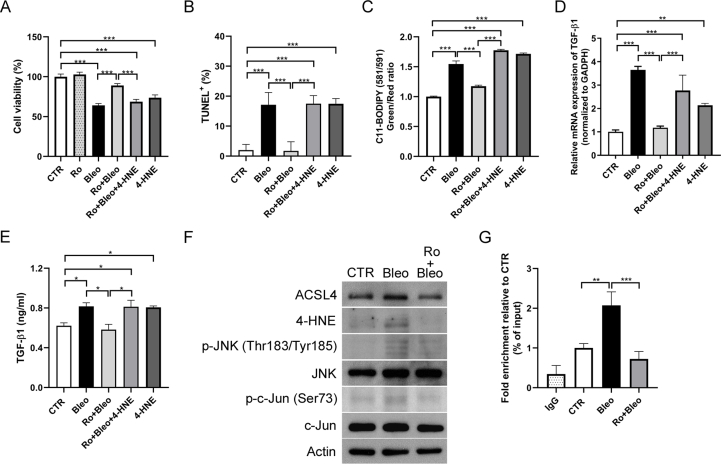
Supplementary Figure S5. **ACSL4 inhibition reduces 4-HNE expression and suppresses TGF-β via JNK/c-Jun activation in A549 cells.** A549 cells were treated with Bleo (10 mU), Ro (5 μM), and/or 4-HNE (40 μM), followed by analysis of: (A) Cell viability, (B) TUNEL assay, (C) Lipid peroxidation, (D) TGF-β mRNA expression, (E) TGF-β levels in the culture medium, (F) western blot, and (G) ChIP-qPCR for c-Jun binding at the TGF-β promoter. Data were normalized to input and expressed relative to the CTR. ∗P < 0.05, ∗∗P < 0.01, ∗∗∗P < 0.001.

**Figure:**
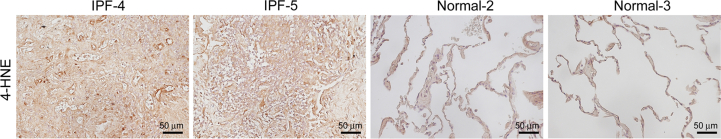
Supplementary Figure S6. **4-HNE expression level were increased in IPF patients.** IHC staining of 4-HNE expression in lung tissue of healthy controls and patients with IPF. Scale bars: 50 μm.

**Figure:**
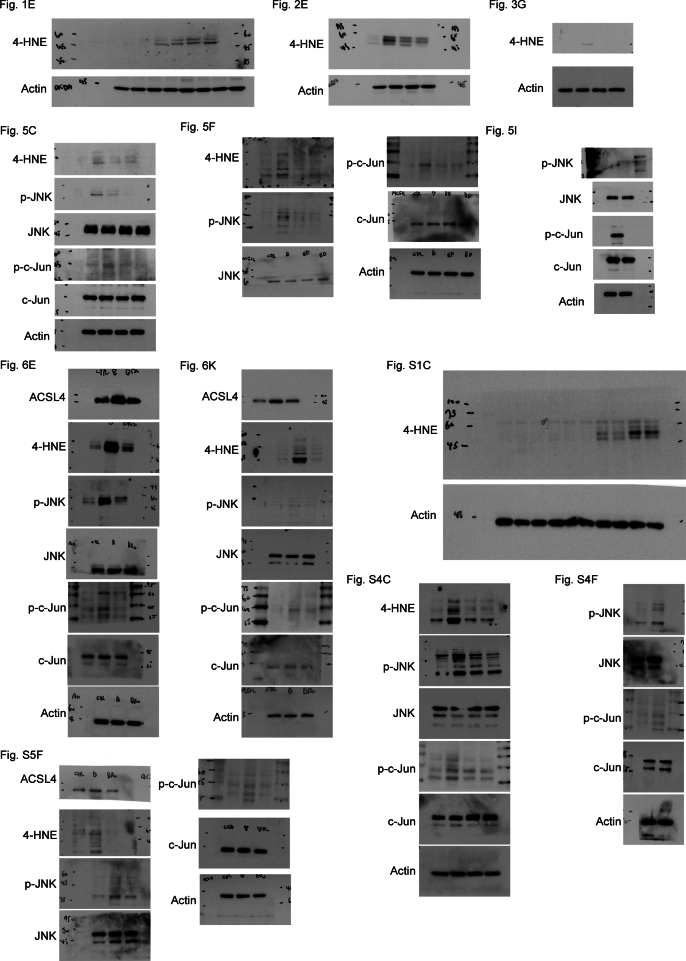
Supplementary Figure S7. Original western blot images.
