# Supplementary material for: Inhibition of the 4-hydroxynonenal-regulated JNK/c-Jun pathway improves bleomycin-induced lung fibrosis
Source: Biomed J. 2025 Oct 10;49(3):100916. doi: 10.1016/j.bj.2025.100916 (PMC13226823; doi:10.1016/j.bj.2025.100916)
Supplement: Multimedia component 1 [file mmc1.docx]

**Supplementary Table S1.** List of primers of qPCR.

| **Gene** | **species** | Forward Primer | Reverse Primer |
| --- | --- | --- | --- |
| TGF-β | Mouse | CACCGGAGAGCCCTGGATA | TCCAACCCAGGTCCTTCCTA |
| MMP2 | Mouse | AACGGTCGGGAATACAGCAG | GTAAACAAGGCTTCATGGGGG |
| MMP9 | Mouse | GCAGAGGCATACTTGTACCG | TGATGTTATGATGGTCCCACTTG |
| TIMP1 | Mouse | CGAGACCACCTTATACCAGCG | ATGACTGGGGTGTAGGCGTA |
| GAPDH | Mouse | AATGTGTCCGTCGTGGATCTG | GCCCAAGATGCCCTTCAGT |
| TGF-β | Human | CCCAGCATCTGCAAAGCTC | GTCAATGTACAGCTGCCGCA' |
| GAPDH | Human | CAACTACATGGTTTACATGTTC | GCCAGTGGACTCCACGAC |

**Supplementary Table S2.** List of primers of ChIP-qPCR.

| **Gene** | **species** | Forward Primer | Reverse Primer |
| --- | --- | --- | --- |
| TGF-β | Mouse | AGGCCTCCTATCGCTCAA | AACAAAGGACAGAGGTGACAG |
| TGF-β | Human | AACCCAGAGAGGAAAAGACT | TGCAGGAAAGGAGAGAGA |
